# Supplementary material for: Agreement between 2017 ACC/AHA Hypertension Clinical Practice Guidelines and Seventh Report of the Joint National Committee Guidelines to Estimate Prevalence of Postmenopausal Hypertension in a Rural Area of Bangladesh: A Cross Sectional Study
Source: Medicina (Kaunas). 2019 Jun 26;55(7):315. doi: 10.3390/medicina55070315 (PMC6681048; doi:10.3390/medicina55070315)
Supplement: Supplementary file 1 [file medicina-55-00315-s001.pdf]

Table S1. Sociodemographic characteristics and risk factors of hypertension among postmenopausal rural women of Bangladesh, $n = 265$.

Characteristics	Mean ± SD	n (%)
**Socio-demographic**		
Age	53.51 ± 7.5	
Age of menarche	13.54 ± 1.78	
Age at menopause	44.83 ± 5.22	
Duration of menopause	8.79 ± 6.45	
Education status		
Illiterate		161 (60.8)
Literate		104 (39.2)
Occupation		
Housewife		239 (90.2)
Service holder		26 (9.8)
Monthly income (BDT)	12279.34 ± 7920.26	
Monthly income groups (BDT)*		
<4906 (low income)		24 (9.1)
4907–19488 (lower-middle income)		187 (70.6)
19489–60252 (upper-middle income)		53 (20)
>60252 (high income)		1 (0.4)
**Hypertension risk factors**		
Current smokers†		4 (1.5)
Current smokeless tobacco users†		119 (44.9)
Physical inactivity		154 (58.1)
Extra salt intake‡		118 (44.5)
Oral contraceptive pill use		91 (34.3)
Diabetes mellitus		53 (20)
Hypercholesterolemia§		68 (25.7)
Central obesity‖		194 (73.2)

*According to the 2006 per-capita gross national income (GNI) and the World Bank calculation. †Smoking/smokeless tobacco use in the past 30 days. ‡Dietary salt intake ≥5 g per day. §Total cholesterol ≥240 mg/dL. ‖Waist hip ratio >0.85.
